# Evaluation on spatiotemporal consistency between solar-induced chlorophyll fluorescence and vegetation indices in grassland ecosystems

**DOI:** 10.1371/journal.pone.0313258

**Published:** 2024-11-15

**Authors:** Longlong Yu, Zhihao Liu, Yangkai Li

**Affiliations:** College of Computer Science, Chengdu University, Chengdu, China; Universidade Federal de Uberlandia, BRAZIL

## Abstract

Monitoring grassland productivity dynamics is essential for understanding the impacts of climate variation and human activities. Solar-induced chlorophyll fluorescence (SIF) has been validated as an effective indicator of gross primary productivity. Satellite-derived vegetation indices (VIs) have long been used as key proxies for vegetation productivity. However, the ability of different VIs to represent grassland productivity in relation to SIF, as well as their spatiotemporal consistency with SIF at various scales, remains unclear. In this study, we systematically compared the performance of the Normalized Difference Vegetation Index (NDVI), the Enhanced Vegetation Index (EVI), and the Near-Infrared Reflectance of Vegetation (NIRv), using SIF as a benchmark in grassland areas of China. Utilizing TROPOMI SIF and MODIS VI datasets from 2018 to 2021, we analyzed the spatial and temporal consistency between VIs and SIF at a monthly scale and 0.05-degree resolution, employing Pearson correlation coefficients, paired-sample t-tests, and two-way Analysis of Variance (ANOVA). The results indicate that NIRv consistently demonstrates a higher capacity to capture variations in SIF compared to EVI and NDVI. In low-elevation areas with high-productivity grasslands, all three vegetation indices exhibit a stronger ability to represent vegetation productivity than in high-elevation areas with low-productivity vegetation types. These findings suggest that, at a monthly and regional spatiotemporal scale, NIRv can serve as a robust complement to SIF in monitoring vegetation productivity dynamics, particularly given the challenges in acquiring high-quality, long-term SIF data.

## Introduction

Grassland vegetation, covering about one-third of the Earth’s land surface, plays a pivotal role as a major component of terrestrial ecosystems, contributing to the equilibrium of land ecosystems, global carbon cycling, climate regulation, and human welfare [1, 2]. Despite its immense significance to the planet and humanity, grassland vegetation is remarkably fragile [2–5]. Primarily distributed in arid and semi-arid regions, grasslands often inhabit transition zones between forests and deserts or occur at high altitudes. Due to their simplified vegetation structure, they are highly sensitive to environmental stressors, rendering grassland ecosystems vulnerable and susceptible to degradation caused by climate fluctuations and human activities [1, 3, 5–8]. Hence, effective monitoring of changes in grassland vegetation dynamics holds undeniable significance. Among these changes, shifts in vegetation productivity stand out as a cornerstone task in tracking vegetation dynamics [6, 8–11].

Remote sensing has emerged as the most potent and indispensable tool for monitoring long-term temporal and large spatial scale variations in vegetation productivity [10, 12, 13]. In the realm of remote sensing for vegetation productivity observation, vegetation indices (VIs) based on spectral characteristics have historically played a pivotal role[9, 13–17]. These indices, such as the Normalized Difference Vegetation Index (NDVI), Enhanced Vegetation Index (EVI), and Near‐Infrared Reflectance of vegetation (NIRv), are widely regarded as primary indicators of vegetation photosynthetic potential and used as proxies of gross primary productivity (GPP) [9, 14, 15, 17–19]. NDVI was initially developed to estimate vegetation greenness, a critical indicator of photosynthetic potential, by capturing the contrast in reflectance between the red and NIR wavelengths [19, 20]. However, NDVI has its shortcomings, including sensitivity to soil background, atmospheric scattering, and susceptibility to saturation in densely vegetated areas [21]. To mitigate these limitations, the Enhanced Vegetation Index (EVI) was introduced. EVI incorporates reflectance in the blue band to correct for aerosol effects, offering improved performance in various conditions [21]. On the other hand, Near-Infrared Vegetation Index (NIRv) is derived from BRDF-corrected NIR reflectance and NDVI. NIRv provides a physical perspective by quantifying the proportion of reflectance attributed to vegetation. Studies have indicated that NIRv exhibits a stronger correlation with Gross Primary Productivity (GPP) compared to NDVI or NIR alone [18, 22]. However, because these vegetation indices indirectly infer vegetation productivity by capturing information related to vegetation greenness and structure, their effectiveness across different spatiotemporal scales and various vegetation types remains uncertain.

Fortunately, the recently retrieved satellite-based solar-induced chlorophyll fluorescence (SIF), which represents the emitted signal from plant chlorophyll molecules following light absorption, has emerged as a novel proxy for gross primary productivity (GPP) [23–29]. This advancement significantly contributes to research focused on monitoring photosynthesis and vegetation productivity [24, 28]. SIF emerges when vegetation re-emits a fraction of the absorbed light energy from photosynthesis in specific spectral bands. This re-emission directly signifies the intensity of vegetation photosynthesis [28]. Now, multiple satellite-retrieved SIF datasets are available, including those from GOSAT, SCIAMACHY, GOME2, OCO-2, and OCO-3 [30–37]. However, these satellite SIF datasets exhibit various data quality limitations, such as low spatiotemporal resolution and sparse strip-like coverage, which restrict their utility as a global GPP proxy [38]. SIF data retrieved from the recently launched Tropospheric Monitoring Instrument (TROPOMI), which offers substantially improved spatial and temporal resolutions (up to 7 km × 3.5 km with daily revisits), holds greater potential than previous satellite SIF data for serving as a proxy for photosynthesis [39–41]. Current global GPP products are based on reanalysis data derived from different models, resulting in datasets of varying quality that often display significant disparities and instability under different scenarios. This variability introduces substantial uncertainty into their results. Therefore, the significance of TROPOMI SIF lies in its potential to serve as a more direct indicator of vegetation GPP, providing an unprecedented opportunity for systematically analyzing VI as a proxy for reflecting vegetation productivity.

In the context of comparing SIF and VI, many studies have introduced both simultaneously in specific cases, analyzing their advantages and disadvantages in parallel, such as conducting phenological analysis, assessing the impact of disaster events, and investigating vegetation-climate change responses [25, 26, 42–47]. However, systematic direct comparisons between VI and SIF are scarce. Some research has revealed the potential complementarity of using both VI, as a structural indicator, and SIF, as a physiological indicator [48]. Nevertheless, even with the high data quality of satellite-observed SIF products, they still face challenges such as low spatiotemporal resolution and limited temporal coverage. Especially when conducting extensive long-term and large-scale analyses, vegetation indices remain indispensable. Therefore, a better understanding of the relationships between VIs and SIF across various biomes and timescales is essential for assessing ecosystem functioning, vegetation productivity, and carbon budgets at regional to global scales using satellite-derived VIs. This clarification can guide the selection of indicators for monitoring vegetation dynamics and contribute to a more comprehensive interpretation of the results. A systematic analysis of the spatiotemporal consistency between SIF and remote sensing VIs for grasslands, which are characterized by vast coverage, simple vegetation structure, and sensitivity to climate change, holds significant applied and scientific value. This analysis enhances our understanding of the performance of commonly used productivity proxies and facilitates the application of these indicators for monitoring grassland productivity dynamics on large spatiotemporal scales.

Building upon these discussions, this study focuses on diverse grassland vegetation types extensively distributed in China. Using TROPOMI SIF as a proxy for vegetation productivity, this research systematically analyzes the spatiotemporal consistency between widely used vegetation indices, particularly NDVI, EVI, and NIRv, and SIF at monthly temporal and regional spatial scales. These scales are commonly employed for large-scale vegetation remote sensing observations and dataset production. Furthermore, the analysis encompasses diverse grassland types, with the aim of providing crucial insights for effective remote sensing observations of dynamic grassland vegetation productivity. Additionally, this study aims to serve as a reference for similar research concerning other vegetation types.

## Materials and methods

### Study area

**Fig 1:**
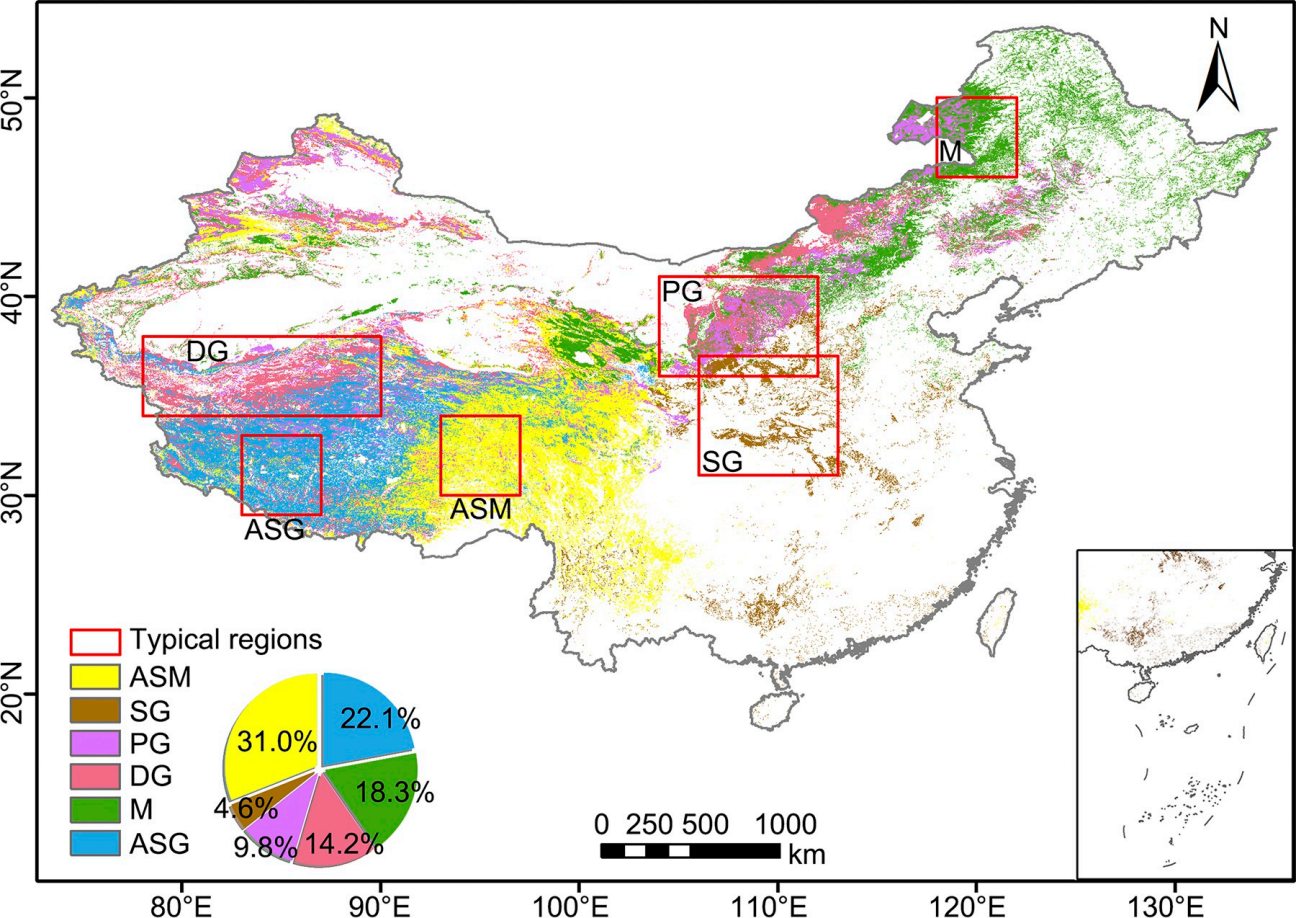
Distribution of grassland vegetation in China. The red boxes represent typical areas of each grassland type that will be used in the time series analysis. (ASM: 30°N-34°N, 93°E-97°E; SG: 31°N-37°N, 106°E-113°E; PG: 36°N-41°N, 104°E-112°E; DG: 34°N-38°N, 78°E-90°E; M: 46°N-50°N, 118°E-122°E; ASG: 29°N-33°N, 83°E-87°E).

This study focuses on the grassland ecosystems in China. With a total area of 3.93 million km2, China’s grasslands constitute nearly 40% of the country’s total land area. These grasslands are primarily distributed in the Qinghai-Tibet Plateau and the northwest region of China, characterized by arid and semi-arid climatic conditions. This vast expanse of grasslands encompasses nine major livestock-husbandry provinces, including Xinjiang, Tibet, Qinghai, Gansu, Inner Mongolia, Ningxia, Shaanxi, Sichuan, and Yunnan. The grassland ecosystems in China face a multitude of challenges due to their sensitivity and vulnerability [2]. Climate change, human activities, and desertification pose significant threats, resulting in varying degrees of degradation across approximately 90% of these grasslands [4, 7]. The grassland vegetation types in China include alpine sub-alpine meadow (ASM), slope grassland (SG), plain grassland (PG), desert grassland (DG), meadow (M), and alpine sub-alpine grassland (ASG). Fig 1 illustrates the distribution of different grassland vegetation types in China, along with the proportion of area occupied by each type. All grassland types in China were included in the subsequent analysis. The red boxes represent typical areas of each grassland type that will be used in the time series analysis, which are selected based on the concentration of target type pixels and the representativeness of the region.

### Datasets

#### Landcover and elevation

The grassland map was derived from the Global Land Cover 2000 (GLC2000) dataset, which features a spatial resolution of 1 km. The dataset was acquired from the following source: http://bioval.jrc.ec.europa.eu/products/glc2000/data_access.php. Subsequently, these data were resampled to a 0.05-degree resolution to match the spatial scale of other datasets. To confirm the land cover type of each 0.05-degree pixel, we employed the MODIS (Moderate Resolution Imaging Spectroradiometer) 0.05-degree annual land cover product-MCD12Q1. Specifically, the MCD12Q1 data spanning from 2018 to 2021 were initially utilized to generate a consistent multi-year 0.05-degree grassland layer. Subsequently, for each 0.05-degree grassland pixel, the detailed grassland type was determined based on the majority rule principle, considering the number of 1 km pixels corresponding to various grassland types within that 0.05-degree pixel. This meticulous approach yielded a 0.05-degree resolution layer encompassing intricate information about grassland vegetation types.

In our study, the 3-arc-second SRTM (SRTM3) DEM V4.1 was used as the elevation data source due to its long-time use and high popularity [49]. It was downloaded from the website (http://earthexplorer.usgs.gov/). The initial spatial resolution is also 1km and was resampled to a 0.05-degree resolution.

#### VI data

The MOD13C2 Version 6 product offers global NDVI and EVI values at a monthly and 0.05-degree scale (https://lpdaac.usgs.gov/products/mod13c2v006/). The MOD13C2 dataset has been widely employed to comprehend and monitor spatiotemporal variations in vegetation, particularly playing a crucial role in investigating vegetation productivity and ecosystem health. The NIRv values were computed from the monthly surface reflectance data of MOD13C2. VIs calculation follows the procedure outlined below.

$$\mathrm{NDVI}=\frac{N-R}{N+R}$$ (1)

$$\mathrm{EVI}=\frac{2.5\times (N-R)}{N+6R-7.5B+1}$$ (2)

NIRv=NDVI×R (3)

where N, R, and B are the reflectance in the near-infrared (NIR), red and blue bands, respectively [18].

#### SIF data

**Fig 2:**
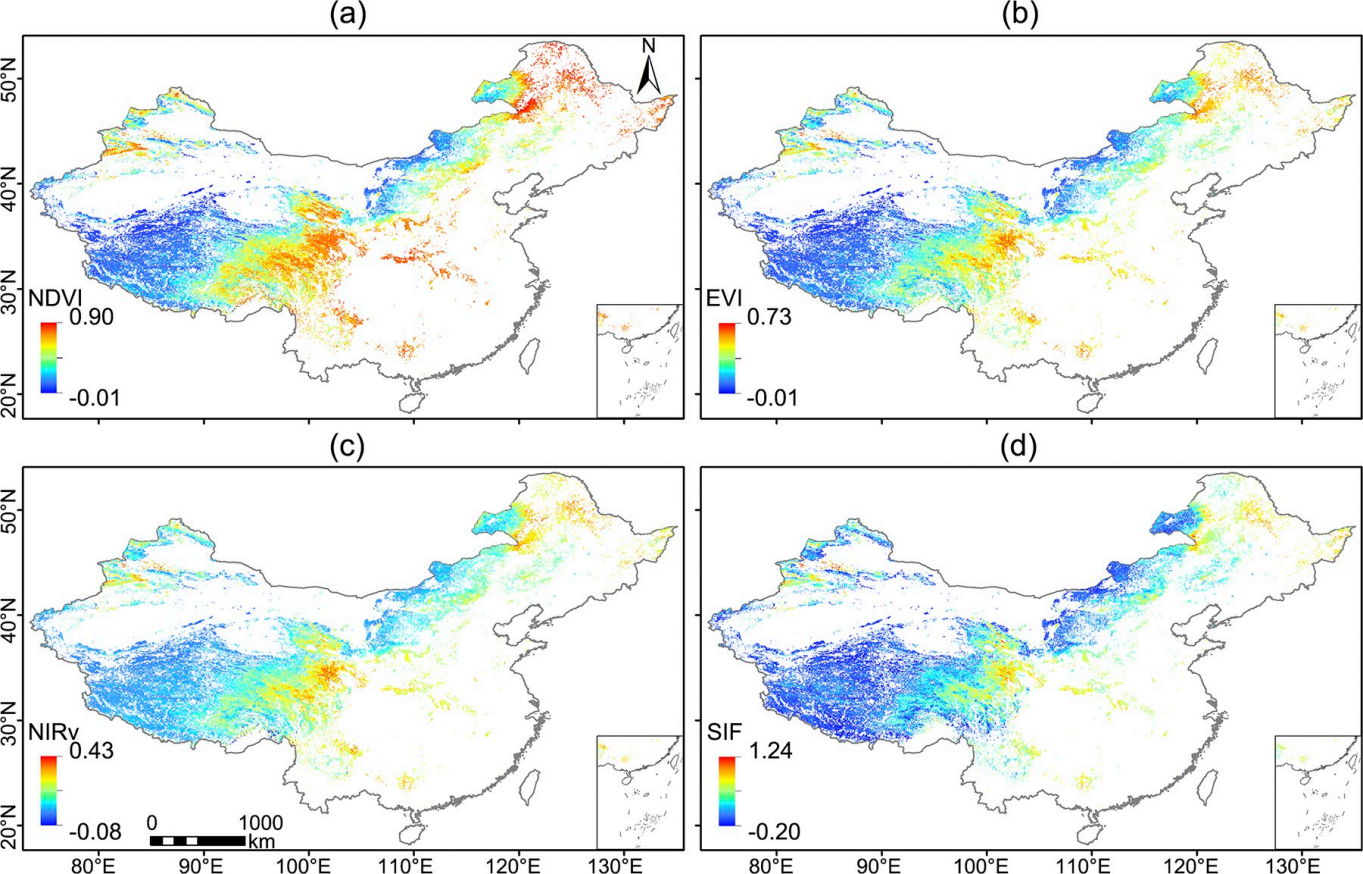
Spatial map of SIF and VIs in Chinese grasslands. (a) NDVI, (b) EVI, (c) NIRv and (d) TROPOMI SIF in July 2020.

We mainly used TROPOMI SIF as a proxy of ecosystem productivity in our analyses. Notably, TROPOMI SIF represents one of the highest-quality datasets among current SIF products, primarily owing to its superior spatiotemporal resolution. TROPOMI SIF, which is available from April in 2018, enables a step change in SIF research, providing unprecedented high spatial and temporal resolution SIF observations that can address many issues related to vegetation productivity [39, 40, 50]. Retrievals of TROPOMI SIF were conducted with a singular value decomposition technique in the window of 743–758 nm and normalized to the SIF at 740 nm. To match the spatiotemporal resolution of the MOD13C2 VIs, the analyses conducted in this study are based on the TROPOMI monthly 0.05-degree gridded data (ftp://fluo.gps.caltech.edu/data/tropomi/). Fig 2 shows spatial maps of MOD13C2 VIs and TROPOMI SIF in July 2020 in Chinese grasslands.

### Spatiotemporal correlation analysis

In accordance with the available data timeframe, the temporal coverage of this study spans from April 2018 to March 2021, encompassing a three-year period. For each 0.05-degree grassland pixel, a comprehensive time series is constructed, comprising monthly values of various indicators, including VIs and SIF, over the course of these three years. Leveraging these derived time series, we employ Pearson correlation coefficients to quantitatively assess the linear relationships between each individual VI and SIF.

The Pearson correlation coefficient (r) describes the direction and strength of the linear relationship, with a value between −1 and 1. We utilized r to depict the correlation between VIs and SIF. The specific calculation formula of r is as follows:

$$r=\frac{\sum_{i=1}^{n}(X_{i}-\bar{X})(Y_{i}-\bar{Y})}{\sqrt{\sum_{i=1}^{n}{\left(X_{i}-\bar{X}\right)}^{2}\;\sum_{i=1}^{n}{\left(Y_{i}-\bar{Y}\right)}^{2}}}$$ (4)

where X and Y represent the two target variables involved in the calculation of the correlation coefficient.

In this study, the above formula is used to calculate the spatiotemporal correlation between SIF and the vegetation indices under evaluation for individual pixels in the region of interest. Specifically, the aligned time series of both indicators at the same pixel location can be used to characterize the temporal correlation between a given VI and SIF at that location. The statistical metrics of all target pixels in the region of interest can then reflect whether this correlation is spatially widespread. All calculations in this study were completed in a Python 3.8 environment, and all data visualization processes were carried out with the assistance of Python 3.8 and ArcGIS 10.6.

This analytical approach facilitates the evaluation of temporal-spatial consistency between VIs and SIF variations at both monthly and regional scales. Furthermore, we endeavor to ascertain the capacity of various vegetation indices to effectively characterize the dynamics of grassland vegetation productivity. Following the computation, a 0.05-degree resolution layer is generated, featuring three correlation coefficients that respectively represent the magnitudes of linear correlation between each of the three vegetation indices and SIF at the pixel level. Subsequently, we engage in a comprehensive visualization of the results and undertake a systematic analysis to explore the intricate inter-play between the three vegetation indices and SIF, thereby providing valuable insights into their temporal-spatial relationships and potential implications for grassland productivity assessment.

### Analysis of variability between vegetation indices and vegetation types

To quantitatively assess the capacity of various VIs to characterize vegetation productivity differences at the monthly scale, we build upon the temporal-spatial correlation analysis with SIF. Building upon the established correlations, we derive the difference layers of correlation coefficients and visualize them, facilitating a direct comparison between different VIs in terms of their performance. Furthermore, utilizing the representative regions of various grassland types as depicted in Fig 1, we compute the time series of normalized values for both the VIs and SIF, focusing on the typical regions. This approach allows us to analyze the temporal trends and mutual relationships between normalized values of VIs and SIF, providing insights into their dynamic interplay. Additionally, we extend our analysis to investigate the impact of different grassland vegetation types and varying altitudes on the correlation between VIs and SIF. By doing so, we aim to offer a comprehensive interpretation of the underlying mechanisms that contribute to these observed differences. Through these endeavors, we strive to enhance our understanding of the unique capabilities of different VIs in capturing vegetation productivity variations and their associations with distinct grassland vegetation types.

### Paired samples t-test

When comparing the differences in correlation between the three vegetation in-dices and SIF, in addition to employing spatial visualization and descriptive statistics, it is imperative to employ inferential statistical methods to ascertain the statistical significance of these differences. This verification aids in determining whether these differences are mere outcomes of random fluctuations or whether they indeed exhibit significant disparities within the larger dataset. To achieve this objective, we have designed paired-sample mean hypothesis tests (t-tests) for experimentation. Specifically, we conduct pairwise comparisons among the three sets of vegetation indices, resulting in a total of three comparisons. This approach will enable us to statistically analyze the differences in correlations between SIF and NDVI, SIF and EVI, as well as SIF and NIRv. In the experiment, we will first calculate the correlation values between each set of vegetation indices and SIF. Subsequently, we will employ paired-sample t-tests to compare each pair of correlation values. The t-tests will assist us in determining whether these correlation differences are statistically significant. We will rely on the outcomes of the t-tests to discern whether there is substantial evidence indicating that these correlation differences are unlikely to be solely attributed to random variability. This inferential statistical analysis method will provide more rigorous evidence to confirm whether the correlation differences between the vegetation indices and SIF are statistically significant in our study, further supporting our research conclusions.

### Two-way ANOVA

To investigate whether the observed spatiotemporal consistency between SIF and VIs is influenced by grassland vegetation types and altitude in a statistically significant manner, we employed a two-way ANOVA. The two-way ANOVA utilizes F-statistics and p-values to indicate the reliability of the conclusions. This method allows us to evaluate not only the main effects of grassland types and altitude but also the potential interaction between these two factors. By analyzing these influences, the ANOVA results will provide robust statistical evidence to determine how effectively each VI reflects variations in productivity across different altitudes and grassland types. The analysis is conducted separately for each VI (i.e., NDVI, EVI, NIRv), requiring a total of three ANOVA tests.

## Results

**Fig 3:**
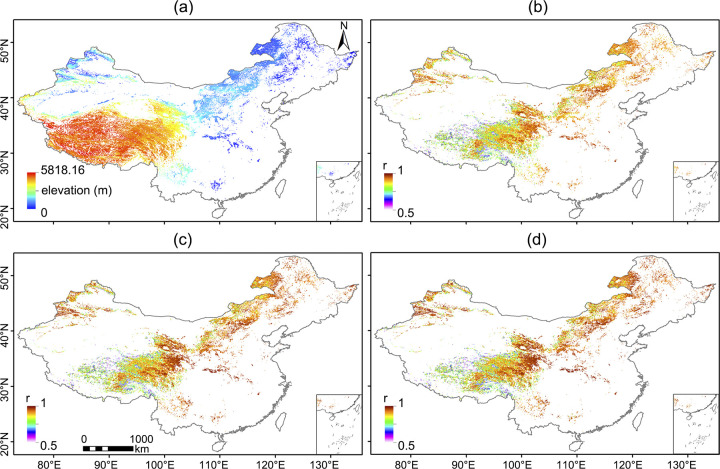
Spatial map of elevation and Pearson correlation coefficient r values between VIs and SIF. (a) SRTM DEM, and r values between (b) NDVI, (c) EVI and (d) NIRv and TROPOMI SIF in Chinese grasslands.

Fig 3 illustrates the spatial visualization of computed correlation coefficients, portraying the spatial distribution of temporal-spatial correlations between various vegetation indices (VIs) and SIF across China’s grassland vegetation. As evident from Fig 3, both EVI and NIRv exhibit stronger temporal-spatial consistency with SIF. However, in this representation, the distinction between EVI and NIRv is less pronounced, indicating that their disparity should be notably smaller than the difference between each of them and NDVI. EVI demonstrates superior resistance to saturation-related issues compared to NDVI, while NIRv tends to better reflect physiological attributes. This observation aligns with the presentation of the results and further substantiates the interplay between the indices and their underlying biological characteristics.

**Fig 4:**
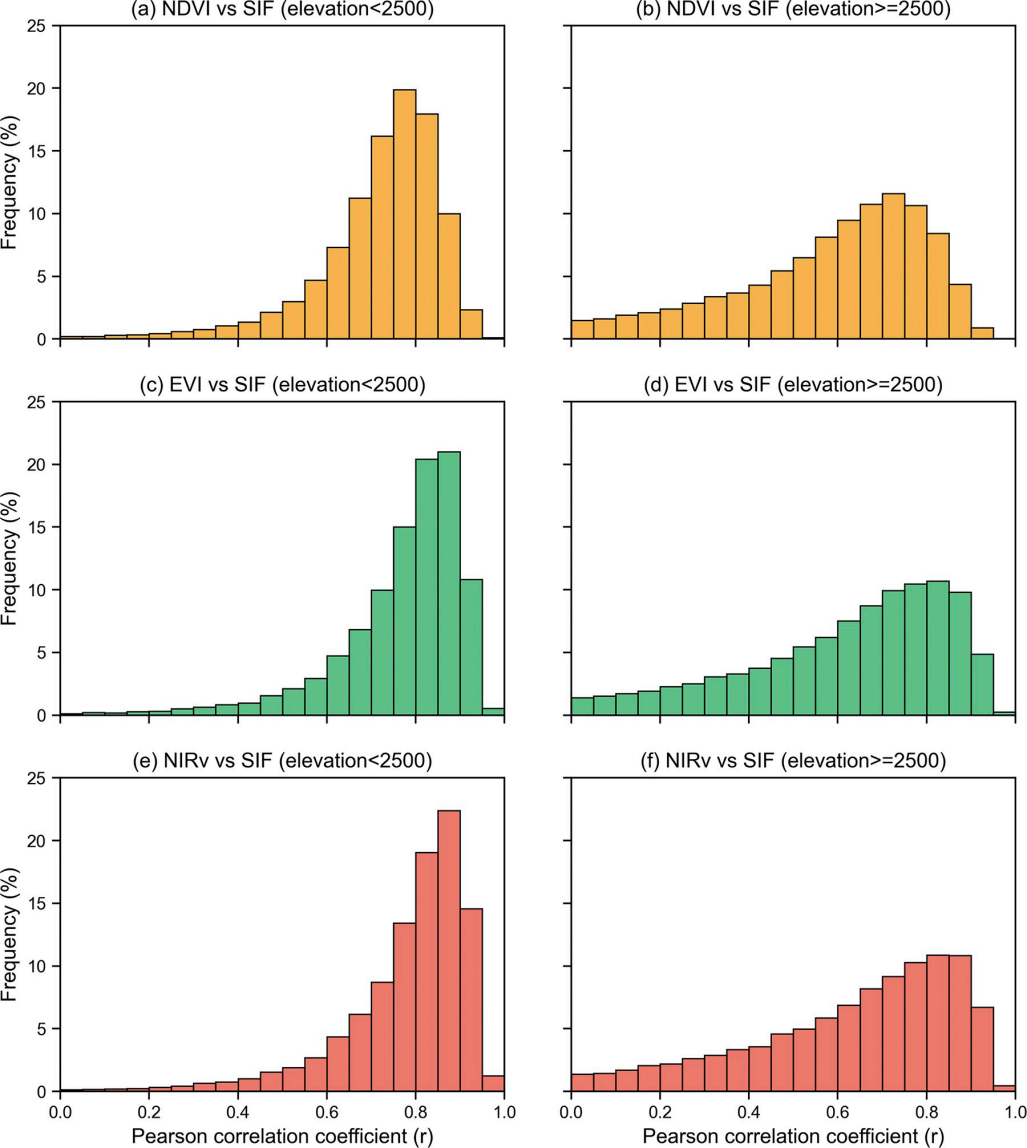
Frequency distribution of r between VIs and SIF in low- and high-altitude grassland.

To provide a more intuitive visualization of the spatiotemporal consistency differences between grassland SIF and various vegetation indices (VIs) at different altitudes, we divided the dataset using an elevation threshold of 2500 meters. We then conducted a statistical analysis of the correlation coefficients and generated a frequency distribution histogram, depicted in Fig 4. Fig 4 reveals several key observations. Firstly, within the lower altitude zones characterized by high productivity vegetation, the distribution of correlation coefficient values between each VI and SIF is concentrated in the higher value range, indicating a stronger temporal-spatial alignment between them. Conversely, in regions with higher altitudes hosting less productive vegetation, the distribution of correlation values is more dispersed, lacking the concentration of higher values. This dispersion signifies weaker spatiotemporal consistency. Additionally, Fig 4 underscores the superior performance of NIRv and EVI over NDVI.

**Fig 5:**
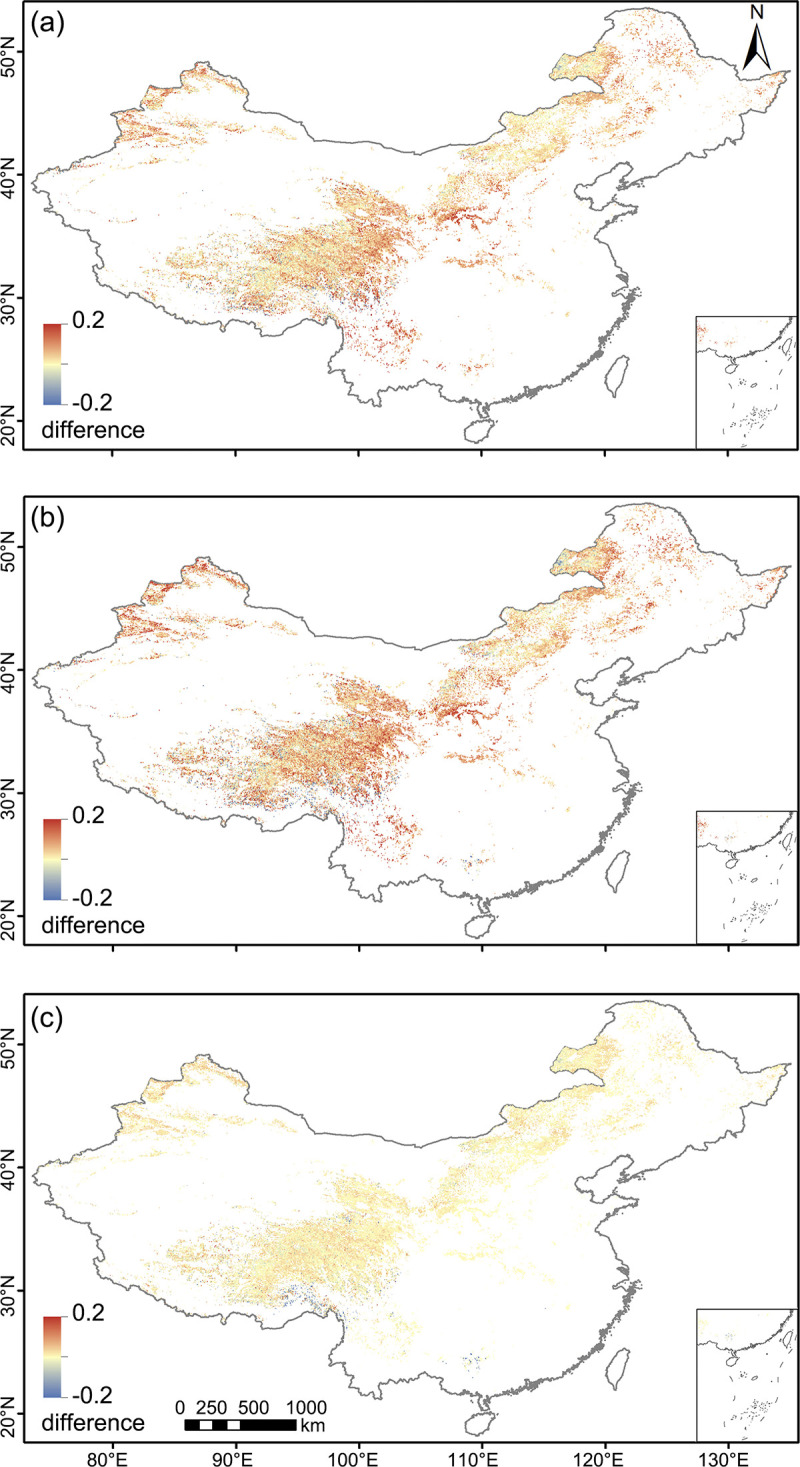
Spatial distribution of r differences. (a) $r_{\left(EVI\;vs\;SIF\right)}-r_{\left(NDVI\;vs\;SIF\right)}$, (b) $r_{\left(NIRv\;vs\;SIF\right)}-r_{\left(NDVI\;vs\;SIF\right)}$, (c) $r_{\left(NIRv\;vs\;SIF\right)}-r_{\left(EVI\;vs\;SIF\right)}$.

Fig 5 displays the spatial distribution of correlation coefficient differences between vegetation indices. This illustration further solidifies our previously obtained conclusions: the superior performance of NIRv and EVI over NDVI and the relatively minor distinction between NIRv and EVI. Moreover, an interesting observation from Fig 5 is that, although the differences are relatively subtle, in the majority of pixels, NIRv tends to outperform EVI.

**Fig 6:**
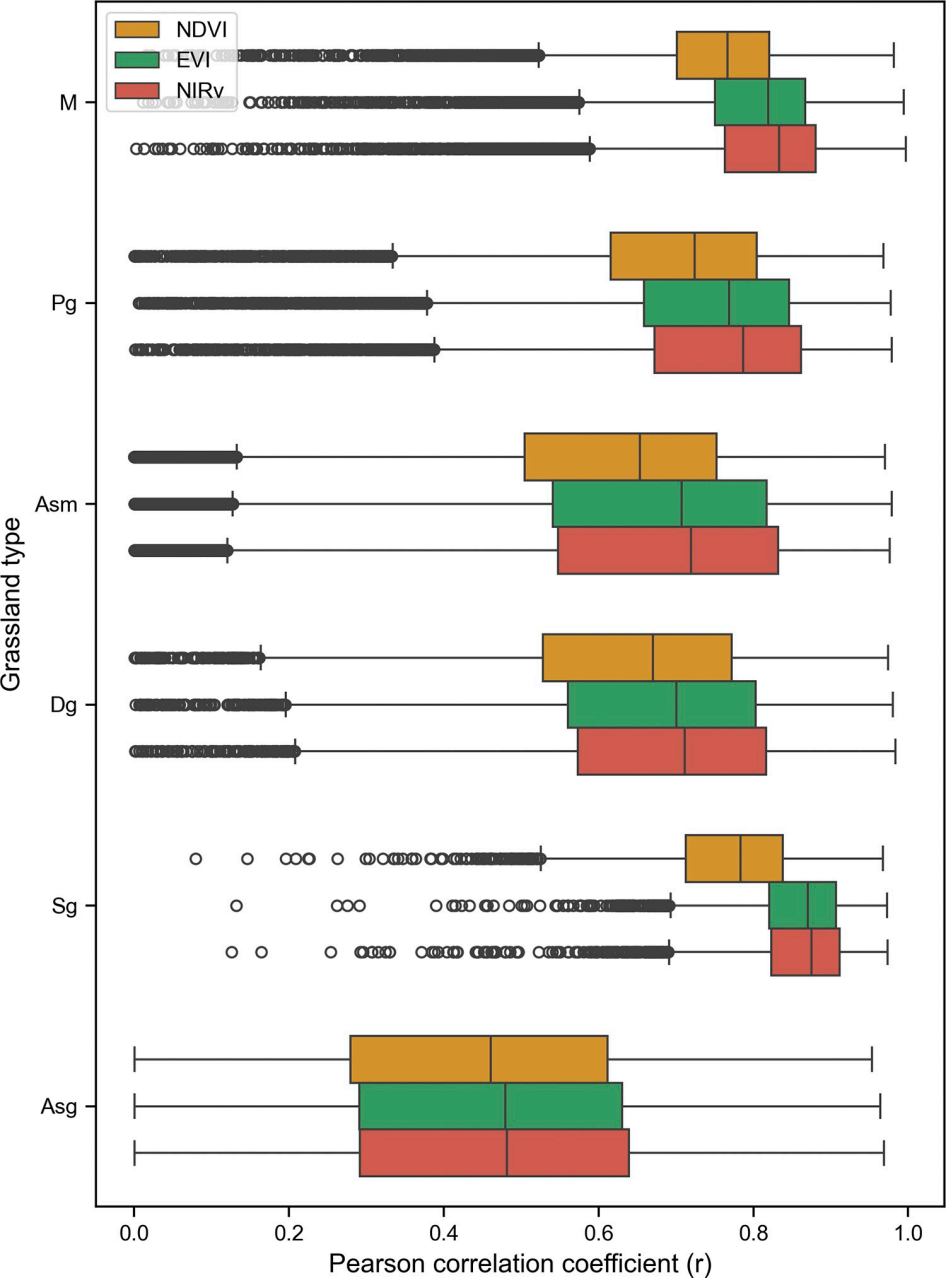
Box plots of r across different grassland types.

In order to further analyze the influence of vegetation types, a statistical analysis was conducted on various vegetation types. Fig 6 presents the statistical box plots of correlation coefficients (r values) between VIs and SIF across different vegetation types. This visual representation highlights the notable disparities among different vegetation types. Notably, Fig 6 also underscores the consistent trend observed earlier: grassland vegetation types in low-altitude areas, such as slope grassland and plain grassland, exhibit stronger spatiotemporal consistency between vegetation indices and SIF, compared to high-altitude regions. Importantly, Fig 6 reveals a consistent pattern across all vegetation types. NIRv consistently demonstrates the highest average performance in terms of spatiotemporal consistency with SIF, followed by EVI, while NDVI exhibits the weakest spatiotemporal alignment with SIF.

**Fig 7:**
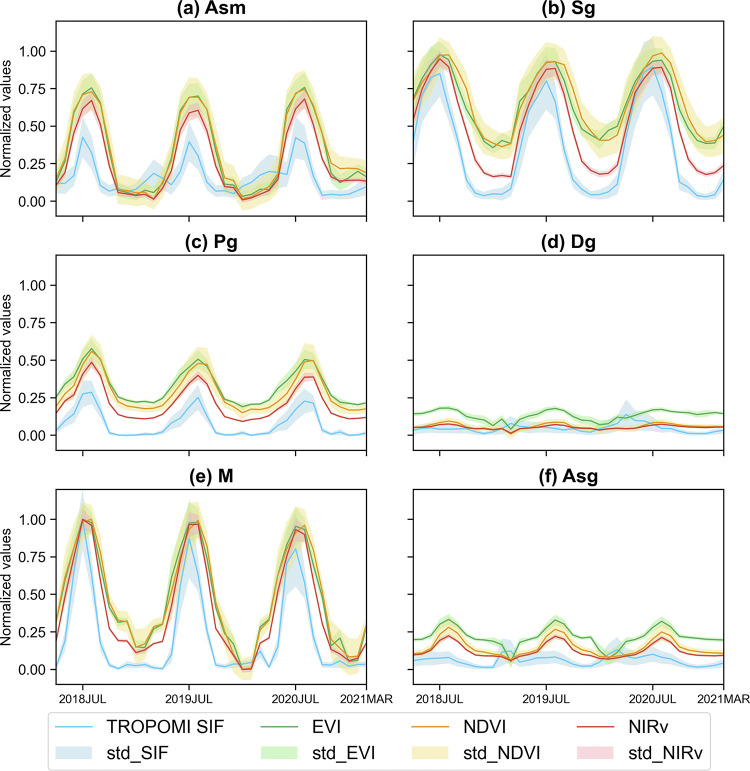
Time series for different grassland vegetation types. The regions used to generate time series are based on the representative box of each vegetation types shown in Fig 1.

Based on the representative regions of each grassland vegetation types shown in Fig 1, we generated the time series plots for different grassland vegetation types as depicted in Fig 7. These values have been normalized to mitigate the influence of units. It is evident that each VI closely tracks the fluctuations of SIF, reflecting their synchrony in response to environmental changes. Notably, among the vegetation in-dices, NIRv exhibits a stronger amplitude of variation that closely follows the changes in SIF. This phenomenon is particularly pronounced in certain regions of the time series curves, and it contributes to the superior performance of NIRv. Furthermore, this trend remains consistent, where the performance of vegetation indices in low-altitude vegetation areas surpasses that in high-altitude regions.

To determine the significance of the spatiotemporal consistency differences between VIs and SIF, paired-sample t-tests were conducted. Table 1 presents the results of these paired-sample t-tests, indicating highly significant differences among NDVI, EVI, and NIRv (p < 0.001). The results from Table 1 strongly support our previous conclusion that NIRv exhibits the highest spatiotemporal consistency with SIF for grassland vegetation, and this relationship is statistically highly significant.

**Table 1:** Results of these paired-sample t-tests.

$H_{1}$ ^a^	T-Statistic	P-value
$r_{\left(EVI\;vs\;SIF\right)}>r_{\left(NDVI\;vs\;SIF\right)}$	149.53	<0.001
$r_{\left(NIRv\;vs\;SIF\right)}>r_{\left(NDVI\;vs\;SIF\right)}$	144.82	<0.001
$r_{\left(NIRv\;vs\;SIF\right)}>r_{\left(EVI\;vs\;SIF\right)}$	58.57	<0.001

^a^*H*_*1*_ represents the alternative hypothesis in the paired sample t-tests. $r_{\left(a\;vs\;b\right)}$ symbolizes the Pearson correlation coefficient between a and b.

Table 2 presents the results of the two-way ANOVA, examining the effects of elevation (Elevation), specific grassland type (Landcover), and their interaction (Elevation × Landcover) on the spatiotemporal consistency between vegetation indices (NDVI, EVI, NIRv) and SIF. The F-statistic (F) and P-value (P) are reported for each factor. Extremely low p-values strongly support our previous findings from descriptive statistical methods, indicating that both grassland type and elevation significantly influence the spatiotemporal correlation between SIF and the vegetation indices. In grassland types with higher productivity levels at lower elevations, the vegetation indices show a markedly better ability to indicate photosynthetic activity. The data in the table provide strong evidence to suggest that elevation, grassland type, and their interaction all have highly significant effects.

**Table 2:** Results of two-way ANOVA on the effects of elevation and landcover.

Factors	Specific VI (vs SIF)	F-statistic	P-value
Elevation	NDVI	15139.53	< 0.001
	EVI	15376.86	< 0.001
	NIRv	15264.08	< 0.001
			
Landcover	NDVI	338.18	< 0.001
	EVI	510.54	< 0.001
	NIRv	451.53	< 0.001
			
Landcover × Elevation (Interaction)	NDVI	66.13	< 0.001
	EVI	150.11	< 0.001
	NIRv	153.24	< 0.001

## Discussion

SIF has been extensively validated as a superior proxy for capturing the intensity of vegetation photosynthesis and serves as an excellent indicator of GPP. In this study, we did not reiterate the well-established role of SIF in GPP characterization. Instead, we focused on a novel perspective. Specifically, we directly compared the most widely used vegetation indices, namely NDVI, EVI, and NIRv, with satellite-derived SIF data (TROPOMI SIF) in the context of grassland vegetation productivity monitoring.

By leveraging Pearson correlation analyses and conducting comprehensive comparisons, we identified distinct variations in the temporal and spatial consistencies between these indices and SIF. Notably, across various temporal and spatial scales, NIRv and EVI consistently exhibited stronger spatiotemporal alignments with SIF than NDVI. Furthermore, in most scenarios, NIRv slightly outperformed EVI in terms of consistency with SIF. This observation is noteworthy as the disparity between NIRv and EVI is notably smaller than that between NDVI and the other indices. The competitive edge of NIRv and EVI can be attributed to their intrinsic advantages over NDVI. EVI incorporates reflectance in the blue band to correct for aerosol effects, offering improved performance in vegetation productivity monitoring. NIRv provides a physical perspective by quantifying the proportion of reflectance attributed to vegetation, and exhibits a stronger correlation with Gross Primary Productivity (GPP). In grassland vegetation regions, the advantage of NIRv in representing vegetation productivity, as indicated by SIF, is partly attributable to the sparse vegetation cover and simple canopy structure present in arid and semi-arid climatic zones. These regions are characterized by dry climates, with minimal cloud cover and rainfall, and are dominated by grassland vegetation with relatively simple canopy structures. Compared to areas with more complex canopies, the process of SIF emission in these regions is less affected by interference from dense canopy layers and atmospheric conditions. However, due to the sparse vegetation cover, a significant portion of the spectral reflectance signal is influenced by the background soil. The performance of reflectance-based VIs in indicating vegetation productivity is therefore highly dependent on their ability to resist interference from soil background signals, and this is precisely where NIRv demonstrates a distinct advantage. These attributes render them particularly valuable for phenological and ecological studies in arid and semi-arid regions, as corroborated by previous research.

Additionally, our study reaffirmed the challenges associated with dynamic monitoring of high-altitude regions and low-productivity vegetation. These zones pose difficulties in obtaining valid SIF observations due to both their limited vegetation productivity and the compromised signal-to-noise ratio inherent in regions with low SIF values. Consequently, vegetation indices’ capability to characterize SIF in these regions diminishes. This is driven by a combination of factors including low productivity and reduced signal quality. The intricate task of effectively monitoring low-productivity vegetation dynamics carries strategic significance. Such efforts are pivotal for comprehending vegetation-climate response mechanisms in the context of global climate change. Notably, these low-productivity vegetation types exhibit heightened sensitivity to external pressures, emphasizing the critical significance of their monitoring. For the effective monitoring of productivity dynamics in low-productivity vegetation types at high altitudes, new vegetation indices and the upcoming satellite sensors specifically designed for SIF monitoring are expected to alleviate this challenge to some extent. Additionally, with the continuous improvement in the quality and quantity of environmental datasets closely related to productivity dynamics, productivity monitoring methods based on terrestrial ecosystem models will also be increasingly driven forward.

Grassland vegetation is primarily distributed in arid and semi-arid climatic zones or high-altitude regions, characterized by simple vegetation structures and low resilience to external disturbances, making it vulnerable to climate change and human activity. At the same time, grasslands represent the most extensive vegetation type globally and play a crucial role in carbon sequestration. Grasslands also cover many biodiversity hotspots, making them essential for maintaining ecological balance on Earth and supporting the sustainable development of livestock farming. Therefore, effectively monitoring grassland vegetation productivity dynamics on large spatiotemporal scales is of significant practical importance for timely and accurate assessment of the impacts of climate change and human activity. Currently, SIF data, which directly reflect vegetation productivity, are severely limited by the lack of satellite sensors specifically designed for SIF monitoring, as well as several data quality bottlenecks, including spatial and temporal resolution, time coverage, data validity, and scale matching issues. This study demonstrates that, at large spatiotemporal scales, NIRv and SIF exhibit strong spatiotemporal consistency in grassland vegetation, highlighting their complementarity and potential interchangeability in grassland productivity monitoring. This finding facilitates the integrated use of multi-source remote sensing data for dynamic monitoring of grassland vegetation productivity.

The temporal scale of this study is monthly, and the spatial scale is 0.05 degrees, which is consistent with many datasets produced for long-time series and large spatial scale analyses, such as MODIS global VI datasets and other common meteorological factor datasets. The conclusions of this paper are limited to grassland vegetation at this temporal and spatial scale. The expression of SIF signals exhibits complex spatiotemporal scale effects, and VI data also carry considerable uncertainty at different scales. Furthermore, SIF and VI signal characteristics may vary significantly across different vegetation types. All of these factors highlight the specificity and limitations of the conclusions at this particular spatiotemporal scale. Detailed analyses specific to other spatiotemporal scales or vegetation types are necessary when dealing with different cases.

## Conclusions

This study employed the widely validated TROPOMI SIF data as a proxy for vegetation productivity and conducted a systematic comparative analysis of three commonly used vegetation indices (NDVI, EVI, and NIRv) with SIF in the grassland regions of China at monthly and 0.05-degree spatial scales. The objective was to assess their performance in characterizing vegetation productivity. The results revealed that, in most cases, NIRv exhibited a higher capacity to capture variations in SIF when compared to EVI and NDVI, with NDVI showing the weakest consistency. Statistical significance was confirmed through paired-sample t-tests. The gap between NIRv and EVI was significantly smaller than the gap between both of them and NDVI. In low-elevation areas with high-productivity grassland, all three vegetation indices exhibited a higher ability to represent vegetation productivity compared to their performance in high-elevation areas with low-productivity vegetation types. These findings suggest that, at a monthly and regional spatiotemporal scale, NIRv is more effective for monitoring vegetation production dynamics in most grassland areas in China. It can serve as a robust complement to SIF, particularly given the limitations associated with acquiring high-quality and long-term SIF data. For low-productive grassland types, future research should focus on developing more effective monitoring methods to evaluate the responses of these fragile vegetation ecosystems to climate change and human activities.

## Supporting information

S1 File Data used to support the findings. The data used to generate the figures and support the key conclusions in this paper are included in this file, along with a detailed explanatory document.
(ZIP)
